# “We think about the quantity more”: factors influencing emerging adults’ food outlet choice in a university food environment, a qualitative enquiry

**DOI:** 10.1186/s12937-022-00801-0

**Published:** 2022-07-30

**Authors:** Daniel Opoku Mensah, Oyinlola Oyebode

**Affiliations:** Warwick Center for Global Health, Division of Health Sciences, Warwick Medical School, The University of Warwick, Coventry, UK

**Keywords:** Food outlet choice, Emerging adults, University food environment, Dyadic interviews, Ghana, Sub-Saharan Africa

## Abstract

**Background:**

In recent decades, the food environment has seen rapid transformation globally, altering food availability and access along with how people interact with the food environment and make food-related choice.

**Objectives & method:**

This explorative study aimed to identify the factors that shape the decision-making process for food outlet choices among emerging adults in a Ghanaian University food environment. The study uses focus group discussions in combination with novel dyadic interviews with best friend pairs. Verbatim transcripts were analysed thematically using NVivo 12.

**Results:**

Drawing on socio-ecological model (SEM) of behaviour, the study used testimony from 46 participants aged 18–25, 47% female, including individuals from major ethnicities and religions in Ghana, and identified three interwoven levels of influence shaping emerging adults’ choices of food outlet. The main factors influencing food outlet choice were identified as food prices, spatial accessibility, budget, and food quantity/satiety with additional factors including hygiene, variety of foods, food quality and taste preferences as well as societal factors such as ambience and peer influence.

**Conclusion:**

Multi-component approaches that combine structural level interventions in food retailing along with individual level components may be effective at changing emerging adult consumption behaviour in SSA, although this needs to be studied.

## Introduction

In recent decades, the food environment has seen rapid transformation globally, altering food availability, accessibility, affordability, and marketing. The role of the food environment has therefore received growing interest in relation to its contribution to food-related behaviours and health outcomes like obesity and nutrition-related non-communicable disease (NR-NCD) [1] as well as implications for sustainability [2]. Many studies exploring associations between local (physical) food environments and consumption behaviour often overlook the fact that neighbourhoods are multi-dimensional and that the effects of an environmental exposure may be modified by another [3] or other non-environmental exposures [4]. Mason et al. [5] and Story et al. [6] argue that social factors like social modelling, social facilitation, impression management and social norms exert important influences on food-related behaviours. Although choosing where to buy from appear to be a simple act, it is intricately connected to individual characteristics and preferences, social/subjective norms and beliefs, and the dynamics of the physical environment.

Almost half of the 20 countries with the fastest rising adult obesity prevalence are located in Africa [7]. The proliferation of highly processed foods (especially sweetened/carbonated beverages, sweetened/salted snacks), and increased presence of ‘Western-style’ fast-food outlets and supermarket chains in Sub-Saharan Africa’s (SSA’s) urban food environment have been implicated in the ongoing nutrition and epidemiologic transitions [8, 9]. Population health research in SSA is challenged to spearhead an understanding of food environment influences on food-related behaviours and health in SSA and identify ways to promote the benefits, prevent the harms of SSA’s changing foodscape and to steer the nutrition transition in the right direction.

Emerging adulthood—18 to 25-year olds [10, 11]—describes the period when individuals establish independence and responsibility for life choices, including food-related behaviours [12, 13]. Previous research has shown that most emerging adults are less likely to meet standard dietary recommendations than other age-groups [14–17]. They engage in high intake of saturated fats and sugar, low fruit and vegetables (FV) intake, tend to display erratic eating behaviours and skip meals [16, 18–21]. During emerging adulthood, living circumstances often change; individuals start living alone, with partners or friends. This may affect their food-related behaviours that may perpetuate into later life [11, 13], affect their own health or that of their partners and/or children or become a model of dietary behaviour for successive generations [22–26]. Longitudinal studies show that healthy eating habits decline when emerging adults transition from living with family to living alone [27, 28] and from adolescence to young adulthood [29]. It has been noted that emerging adults may lack the skills to negotiate the multiple constraints posed by the current food environment causing them to often settle on unhealthy options in their food-related decisions [30–32]. This period presents an opportunity to influence the adoption of healthy lifestyles, including dietary behaviours for holistic benefits. Globally, SSA has the youngest population and this age-group is projected to double by 2050 [33]. To advance in combating dietary risk factors associated with obesity and NR-NCDs, an understanding of SSA’s foodscape influences on its emerging adults’ food-related behaviours is an important pre-condition.

Socio-ecological models (SEM) of health behaviour offer the opportunity to capture a more holistic picture of the multiple influences on a particular health behaviour [34]. It captures the interrelationships between the physical environment, the social environment, and the individuals who live in, shape, and respond to those environments [35]. This paper reports the results of applying the SEM to explore emerging adults’ interaction with their university foodscape in their daily food shopping decisions in urban Ghana.

## Participants and methods

This study, using data from both best friend pair interviews (BFPI) and focus group discussions (FGD), is part of a wider project exploring the impact of the urban food environment in SSA on food-related behaviours of educated urbanites. The study is reported following the Consolidated Criteria for Reporting Qualitative Research (COREQ).

### Recruitment

The research recruited students from the University of Ghana campus. Participants were recruited from all colleges of the University through poster advertisements on residential, departmental and faculty notice boards as well as on social media platforms and through in-person invitations. Participation was voluntary and students at all levels of study were eligible to participate as long they were aged 18 to 25 years, the average age bracket for most university students in Ghana.

Focus groups consisted of 3–8 participants. For best friend pair interviews, a participant and one best friend only were eligible to participate. In this study, a best friend (or close friend) was defined as “a person within participants’ own age group who they knew very well; with whom they met regularly (at least, a couple of times per week), engaged in activities with, ‘hang out’, and/or had fun or ‘chilled out’ with, and with whom they shared emotional or difficult moments” [36]. This must be a member of the university community, but not necessarily from the same faculty, department, or hall of residence. Eligible participants who expressed interest were given study information packs, including consent forms and a brief demographic questionnaire.

### Data collection

A semi-structured approach was adopted with a topic guide used to inform the interviews and discussions. The topic guide was developed iteratively and piloted in a BFPI and an FGD with students from the study campus, data from which were later included in the final analysis as no significant modifications were subsequently made to the topic guide. This topic guide was developed to explore determinants relating to emerging adults’ food-outlet choices and eating/food-related behaviours. It also invited emerging adults’ opinions on what changes to the food environment would support them to undertake healthy/sustainable food-related behaviours. Data collection was conducted by the first author (DOM), who had previous experience and training in qualitative interviewing. Research assistants took turns in assisting DOM to take field notes and audio-record interviews and FGDs. FGDs and BFPIs were conducted within the University campus and at participants’ convenience to minimise discomfort or distress. The study aimed for a minimum sample size of 48 using the principles of data saturation [37] and maximum variation sampling [38], along with DOM’s time constraints. Basic socio-demographic information was collected from the participants through self-report. Data collection proceeded between November 2019 and March 2020. Each participant was given a ballpoint pen and airtime voucher in compensation for their time.

### Analysis

Verbatim transcripts were analysed thematically after Braun & Clarke [39] by DOM after reading transcripts many times to familiarise with the data. Transcripts were initially coded line by line using NVivo version 12 [40] and then indexed into data tables to create descriptive themes. Descriptive themes were compared to identify patterns in order to generate analytical themes. Based on the pragmatic double coding process as described by Barbour [41], emergent themes were refined iteratively based on discussions with other members of the research team. Themes were presented to participants for authenticity checking.

## Results

In total, 46 emerging adults participated in eight BFPIs and 7 FGDs, lasting 60–75 minutes respectively. Fieldwork was curtailed due to COVID-19 restrictions on campus, but saturation was achieved at 46 participants. Full demographic details are presented in Table 1. All interviews and FGDs were conducted in person on the University campus in an enclosed meeting room or at participants’ residence (in one BFPI).

**Table 1:** Characteristics of participants

Variable		Number	Percentage
Gender	Female	21	46.7
	Male	25	53.3
Age range	18 to 24 (mean 21.2)	46	100
Nationality	Ghanaian	46	100
Ethnicity	Akan	17	37.0
	Ewe	14	30.4
	Others	12	26.1
	Prefer not to say	3	6.5
Level of study	Year 1	3	6.5
	Year 2	11	23.9
	Year 3	21	45.7
	Year 4	10	21.7
	Postgraduate (PG)	1	2.2
Religion	Christian	39	84.
	Muslim	5	10.8
	Not religious	1	2.2
	Prefer not to say	1	2.2
College	College of Basic and Applied Sciences	7	15.2
	College of Humanities	29	63.0
	College of Education	5	10.9
	College of Health Sciences	5	10.9
< 18.5	Underweight	9	19.6
18.5–24.9	Normal weight	29	63.0
25.0–29.9	Overweight	6	13.0
30.0–34.9	Obese	2	4.4
Accommodation type	Family/Guardian	1	2.2
	Private Hotel	12	26.1
	University-managed Hostel	33	71.7

### Themes: determinants of food-outlet choice

#### Environmental factors

##### Price

Most students (*n* = 32) considered the cost as the principal factor in choosing an outlet for food.

“...I will also say the same thing. So anytime I'm going to buy food, there are two things I look at: the cost of the food, and then the brand name or the recognition” (R1, FGD 6. Male).

“Affordability is the most important for me. Because I know I can’t afford. So I just either go to Bush Canteen, [or] Night Market...” (R2, FGD 2. Male).

Students were minded to shop at outlets that offered the best fit with their status as students and their income/ socio-economic status. Some food outlets appeared to be the preserve of University staff and were described by students as not being in their “league” as they were thought to be “really expensive.”

“Ok so I think basically because of the cost or the price of their foods. Again, to me basically I think its because of the price or the cost their foods that they serve or they sell.” (R1, FGD 6. Male).

Some students sacrificed convenience in order to get the best deals. According to respondent accounts, a small section of students travelled long distances to buy from certain outlets because of affordability. Such students avoided proximal outlets, including those within their hostels or halls of residence, to patronise those that offered variety and value for their money even though they were usually far off.

“I don’t think distance really matters because sometimes you see people whose hostels or halls are like let me say about 2 kilometres or like people who are not even on campus coming all the way to the Night Market just to buy food. So the motivation is the variety of foods and then the price.” (R2, BFPI 6. Female).

Convenience shops within halls and hostels, and campus supermarkets were viewed as expensive outlets unsuitable for “bulk food shopping.” They were mostly used in emergencies, to buy snacks, beverages, and water.

##### Spatial accessibility

Issues relating to the convenience of accessing food outlets were frequently reported by students. When hungry, most students would want to buy from the closest food outlet. Convenience was even more important during the exam period. Walking long distances to the bigger food outlets with a wider variety was something many students did not want to do. In many cases, students used food joints on their commute between their classrooms and hall/hostel of residence. Otherwise, they would only walk if the distance to the food outlet was “walkable far.”

“… most of the time its rice I eat. And then as he said, sometimes if I'm hungry, I don't really want to walk far to go and get something....And most of the time I go to Night Market because I see Bush Canteen as a little far from me.” (R3, FGD 5. Male).

There was an internal conflict in emerging adults when the most convenient food outlet was seen as more expensive or to have less variety of food. However, many students settled on those food options closest to them, despite this conflict, including the many “indomie” instant noodles joints dotted around the halls of residence, and savoury and sugar-sweetened snacks which were the most popular in grocery stores within hostels and halls of residence.

“… I think about the closeness. Sometimes even though I’ve budgeted my money for the week, I’ll still come here [dining hall in their hall of residence] because I feel it’s hard work to walk all the way…” (R2, FGD 2. Male).

“Some people consider distance. For Indomie, they're very near to us… two, three steps, we're there. You buy and then you just go back...” (R2, FGD 4. Female).

The few students that used private cars on campus also shared a similar view. Although they were the group that would most often buy prepared food from outlets outside of campus, they wanted to eat from places closer to the campus and other places they would avoid traffic delays.

“And then the nearness or the location of wherever I want to eat. That one too is important. Because if I'm really hungry I don't want somewhere far that I'll be in traffic and so I'll just look for somewhere close to campus.” (R2, BFPI 2. Female).

##### Hygiene

Many emerging adults were concerned about hygiene etiquette of caterers/vendors and the environment in which food was prepared.

“Your appearance, how you appear towards your customers. For instance, if you’re woman and you cover your hair with something when you’re selling the food, I would like to buy from you than someone who always leaves her hair.” (R2, BFPI 5. Female).

“You see with big restaurants you don't see where they make the food. Unfortunately for us, with our places, especially Bush Canteen and the Night Market, you see where they make the food. For Bush Canteen for instance where they sell, it's ok. I'm not going to say it's dirty. Or even Night Market. Like the environment is not really dirty. When you look at the back, where they actually do some preparation, that's the place that is nasty to me...” (R2, FGD 5. Female).

Some students believed that certain dishes, especially soupy meals, required careful handling during preparation. They did not think that some food outlets were hygienic enough to safely manage the preparation of those meals, considering that many vendors of such foods did not have properly engineered kitchens and used ‘makeshift’ arrangements. Soupy dishes like fufu with soup and banku with soup were the food options mostly cited by emerging adults as requiring the most hygienic environment.

Some students also considered whether food outlets had a hygienic seating area where they could sit comfortably to eat.

Based on hygiene concerns, a small minority of the students did not buy food from campus food outlets and preferred to prepare their own food or bring food from their family or relations’ homes if possible.

“Yes. 'cause I don't really like buying around 'cause of like the hygiene and everything. Like I don't really like how they cook on campus; most of these Bush Canteen... and the Night Market vendors. Ha! I don't like how they cook and how like there are flies and... like everything around. Is not appetizing to me. So I mostly cook my breakfast and eat. Then I can cook maybe jollof and keep some in the fridge. So when I come I'll heat it.” (R4, FGD 5. Female).

There was evidence of students who had no option apart from buying from campus food outlets even though they expressed concerns about hygiene.

##### Variety of foods on offer

Most students considered the variety of foods offered when deciding where to eat. According to emerging adults, this meant they were sure to find some food they wanted to eat, compared to visiting places that offered only one or two types of dishes where they may be disappointed after walking “all the way”.

“Ok, is because over there you can find all manner of foods there. Like different types of food…” (R2, BFPI 6. Female).

“… I also think it's because there are variety of foods there as compared to the other places. 'cause if you go to Night Market, for instance, you get rice, you get banku, fufu, like yeah, varieties.” (R3, FGD 1. Male).

Some students prioritised outlets that offered fruit options as part of the variety of foods on offer or outlets that included a wide range of local dishes.

“… I think people also prefer those places or I prefer going there because they also sell other things some of the places in the halls do not sell. Like fruits, bananas and egg, most eateries in the halls do not sell those. And for someone like me who like enjoys having bananas, I definitely will want to go there and then buy.” (R1, FGD 2. Female).

“I think students also prefer those ones because there are more local foods at the Night Market and Bush Canteen. Because with the dining halls and the... halls, they don't have the variety in terms of the local food. They just have a few. Maybe the popular ones like the banku and fufu. But the Bush Canteen and the Night Market, you may getkenkey with pepper, you can get the gari and beans, you can also get TZ [tuo-zaafi].” (R1, FGD 4. Female).

##### Hours of operation

Popular food outlets were open round the clock.

“…food is never finished. There's always food there. They close but quite late. Probably at 12 midnight or so.” (R2, BFPI 2. Female).

“And I think because is 24/7, almost 18 out of 24 hours. It's almost 99 point something [percent], they run from morning till Midnight.” (R1, BFPI 4. Male).

From respondents’ account, many students stayed up late or studied into the night. Such students therefore considered outlets from which they could access food when they felt hungry even at night. The language used by students who expressed this need used the idea that they placed value on a food-outlet being reliable around the clock.

“And the time too. The time Night Market operates is somehow better and they stay much deeper in the night. So, at any time of the day you go there you'll find something to buy.” (R1, FGD 1. Male).

##### Food vendor attitude

Students were also particular about how food vendors treated them. They considered the attitude of food attendants in deciding where to buy their food. Whether or not vendors were polite in their speech or respectful in their attitude towards students determined students’ continued patronage.

“I also consider attitude. Like the person selling the food. Your appearance, how you appear towards your customers and how you talk to people. Yes, so if you’re someone who always frowns, I won’t buy.” (R2, BFPI 5. Female).

“And then the servers’ attitude. I’ve actually stopped buying food at some places in Night Market because of their attitude. Sometimes they make you feel like they don’t need your money, but they have also forgotten that without us they can’t also survive. It’s like a symbiotic relationship.” (R1, BFPI 3. Female).

One respondent reported how a privately managed hostel dismissed a food outlet operator due to constant complaints from students about her conduct.

“There was this was lady, she used to treat everyone badly. Like she's so rude. So they just sacked. Like that's how they regulate it. So if you have a complaint and you tell the hall or hostel managers, they'll do something about it.” (R4, FGD 5. Female).

Although other students indicated that such dismissals and regulation did not usually happen with in university-managed facilities including those in halls of residence.

##### Quality, including taste and freshness

A section of the emerging adults also considered the quality of the food served at the various outlets in deciding which outlet to buy from. For some students quality was assessed by taste.

“And sometimes the quality because ehm, the food they sell at Bush Canteen, example ‘gobe’ [gari and beans], is way way nicer than they sell here at Maxi Catering Service.” (R3, FGD 2. Female).

Some emerging adults highlighted they would sacrifice price for taste once they were confident the food from the food joint would offer them the taste they preferred.

“The taste comes before the prices because sometimes I don't really give too much for a price unless its outrageously high. If I know your food will give me like that taste and everything, no matter the price if is not very high, I can manage.” (R6, FGD 1. Male).

Other young people did not only prioritise taste over price, they were willing to also travel long distances to food outlets where they were assured of that preferred good taste. In one instance, a student reported that the transport cost to one of her favourite food joints was three times the cost of the food itself, but always preferred to buy from that outlet because of taste.

“The taste of the food. I don't really care about the distance. I take Bolt [taxi] in and out for like 30 cedis. But because that’s the waakye I want, I'll take it [taxi] to go and buy the waakye which won't even cost more than 10 cedis. The taste really really matters.” (R3, FGD 3. Female).

For other students, they would not buy vegetables and other food items from campus food outlets due to concerns with the quality of vegetables usually available. According to respondents, vegetables and other food items available at the food outlets on campus were usually not fresh. Therefore, to find fresh vegetables or foodstuffs they preferred to buy from outside of the University campus.

#### Societal factors

##### Ambience

A section of the respondents also considered the ambience of the food outlet in choosing where to eat from. Emerging adults distinguished two atmospheres at food outlets within the university foodscape namely, (i) “neat restaurant” or “continental” setting and (ii) the “local setting” or “typical African market experience”. From respondents’ account, the “neat restaurant” or “continental” setting was the formal environment where they felt one had to observe table manners or eating etiquette to avoid embarrassment. While it was reported that this made students feel “rigid” and “bound”, emerging adults expressed preference for the “typical African market” ambience where they were free to make “noise” and behave freely with their friends. They did not have to follow any formal rules and table etiquette.

“…when you go over there to Bush Canteen and Night Market, there is this unity and noise and everything ‘cause that is why my friends and I would want to go and then eat. But then I can’t go to these restaurants and go and sit or even talk because you have to observe food etiquette but over there its more or less like we are in our own…” (R3, FGD 2. Female).

“I think that she’s talking about like the typical African market experience sort of. ‘cause then when you come here, even though they sell local dishes, it’s like continental because you have to join a queue, pay up, then they will give you the receipt. It’s not like there [Night Market and Bush Canteen] where you get to get the typical African experience with the noise, the local stuffs...” (R1, FGD 2. Female).

“And I get the sense of like a local or you feel more free let me say, because of the local setting and all that.” (R1, FGD 4. Female).

Other young people pointed out the importance of privacy at the seating or dining area of food outlets. According to respondents, when the dining area was not “enclosed” it created an uncomfortable atmosphere as all passersby and other customers would be watching while they ate.

“Sometimes too the environment where…you get to sit and eat. Sometimes you go and its chocked or sometimes it’s like too open. If you’re eating everybody will be watching you. But for some places its quite enclosed, so you can feel free and eat over there. That’s why most at times I usually go to Bush Canteen.” (R1, BFPI 5. Female).

Although young people expressed their preference for the traditional African restaurant atmosphere, they also considered some level of privacy at the dining areas of eateries in deciding where to eat from. These factors, according to them, together created the comfortable ambience within which they could “feel free and eat.”

##### Peer influence

Another determinant of food outlet choice reported by young people had to do with social modelling, whereby other people’s choice served as a guide for where young people bought their food. Friends and roommates influenced young people’s food outlet choice. Based on the testimonial of peers, students would want to patronise certain food outlets to verify their friends’ endorsement.

“...sometimes our friends recommend a particular to us. So you want to also taste the food. So a friend went to buy something from Night Market and was like there are two people who sell kenkey and there's one that people like and there's one that people don't like. Those kinds of experiences will lure me to also buy food from those places…” (R3, FGD 1. Male).

From respondent accounts, other young people got to know about new food-outlets through their peers. Following such recommendations, some students would try food at a new food-outlet.

“And also based on other people's testimonial, if I hear that ok there's this new place, the food is really good, they treat people well and everything, I'll definitely want to try it.” (R1, FGD 2. Male).

##### The occasion

Social gatherings also influenced where young people ate. This was reported by a small number of emerging adults. During special occasions such as birthdays, students did not eat at their usual locations.

“…if it's outside campus then its the occasion or the event, first of all, what are we celebrating is going to determine the location; where we should go.” (R6 FGD 1. Male).

“And then the last one is the occasion. So if it's a special occasion like maybe... ok, maybe if it's a night out with friends or a birthday party or something like that, that'll also decide where I would go and eat.” (R2, FGD 1. Female).

Students reported that they came together with friends to celebrate such occasions and therefore would usually require venues large enough to accommodate their friends and guests.

“...everything she said is like me. I look at the occasion and event. Like my birthday, for instance, I cannot go to my Night Market to buy... beans. I mean, everybody knows you're celebrating birthday and all you went to do; buy just beans is, I mean, weird. But on your birthday, at least you have to glorify God through some kind of get together or something.” (R4, FGD 1. Female).

#### Intrapersonal factors

##### Budget

One of the common personal level factors reported by young people as influencing their choice was how much and how often they received remittances. It was, in most cases, on the top of participants’ list of choice determinants and usually the decisive factor for using one outlet over the other. They prioritised outlets that enabled them to spend within their means. According to students on low budgets, they would usually not join their “rich friends” to eat from the same food-outlet. This tended to influence many young people to focus on the need for volume and satiety in order to defer another expenditure on food.

“…it depends on your budget and how much you receive that will really affect where you buy your food from. If I receive like 100 cedis a month, I’ll certainly buy food always from the bush path because I know I can’t afford this. So, I think the better your income, the better your feeding. Something like that. So your money talks about everything...” (R1, BFPI 3. Female).”

For those that preferred to buy from food outlets outside of the University campus, they considered the cost of the food itself and costs relating to transportation to and from the food outlet where their preferred food was sold.

“…So if it's this food that I want to eat, how much am I willing to spend on the fufu that I want to eat? And then the money too in terms of transport because I drive. So then I'm thinking about if I want to go to somewhere [off-campus] to go and eat fufu, like my petrol. So is it worth burning 20 cedis worth of petrol to go and eat 50 cedis worth of food and coming back?...” (R1, BFPI 2. Female).

From the account of emerging adults who preferred to prepare their own food to cut cost, the cost of buying from an outlet included the cost of delivering the food to them.

“Because if I don't have enough money, why would I want to eat out? Because if they don't do deliveries, I'll have to go myself. And if they do deliveries, I'll have to pay for it; the delivery.” (R2, BFPI 2. Female).

##### Preferences

According to some emerging adults, the food they wanted to eat was a key determinant of where they bought their food from. Students reported that because they could not prepare certain dishes on campus, whenever they craved to eat those foods, they had to buy them from a food outlet.

“So I first consider what I want to eat, so its kinda like cravings but not really craving but like maybe I want to eat fufu but I can't do fufu on campus. So now the next thing that I'll think about is where will I go and eat this fufu?...” (R1, BFPI 2. Female).

##### Quantity/satiety

Most students considered the quantity of food per price they paid in choosing where to buy their food from. Considering that most students prioritised satiety and affordability, how much food they were given in exchange for their money featured prominently. In many cases, the distance between the hall of residence and the food outlet that offered “quantity” did not matter.

“...I also consider the quantity of the food, especially when I'm buying from the canteen. I prefer coming to buy banku from the Night Market than Basket Market at Commonwealth [hall of residence]. Yes, so I sometimes come here to buy the food and I'll carry it back to eat. I prefer that to buying from Commonwealth although that’s my hall and its closer to me but I consider the quantity...” (R5, FGD 5. Male).

“…most of the food vendors have a perception that students have money or something like that. So mostly the food is costly. So when I'm to buy food, I go to a food vendor I know I'll get the quantity that matches the cost.” (R1, FGD 1. Male).

## Discussion

**Fig. 1:**
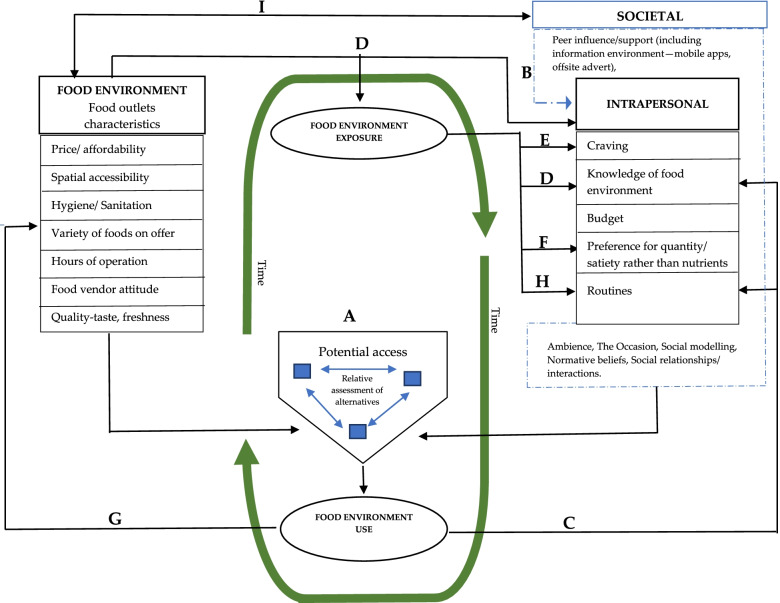
Schematic representation of the relationships among the main themes: **A** Students assessing the potential food outlet options (considering distance to outlet, opening hours, access to cookware, time needed to prepare or procure food); **B** information environment [students mostly relied on their social network—friends, colleagues, and roommates—complimented by information from phone-based applications) for information on available food outlets; followed by knowledge from their own past experiences of using the foodscape (**C**) and exposure to outlets they encountered in their daily classroom-residence journeys (**D**)]. **E** Cravings (food environment exposure influenced individuals to form intentions to eat). Preference for quantity/satiety (**F**) due to mismatch between environmental level and personal level factors, leading to routine use of outlet(s) offering more calories for price (**H**). The prevailing university food environment characteristics appeared to be a product of continuous patronage by students over the years (**G**)

To our knowledge, this is the first study to apply the SEM to explore the socio-ecological dynamics of food shopping among emerging adults in an urban foodscape in the low- and middle-income country setting. The study identified three interwoven levels of influence shaping emerging adults’ choices of food outlet: intrapersonal, societal, and environmental. The main factors influencing food outlet choice were identified as price, spatial accessibility, budget, quantity/satiety, and preferences. The findings shed light on the complexities of the decision-making process for food outlet choices and on the influences of the food environment on dietary behaviours (and vice-versa). Figure 1, a model adapted from Clary et al. [42] is used to discuss the study findings and describe the interrelationships among the levels of influence and sub-themes reported under the results subheading.

The findings show that students usually weighted characteristics of food outlet options available to them against their relevant criteria to settle on the option chosen. Food-related choices have largely been studied through a utility-maximization lens [43–45]. Drawing on this rationale, individuals are viewed as rational agents assessing food outlets against five facets of accessibility—including availability, spatial accessibility, affordability, convenience and acceptability—to settle on using the best available option [42, 46, 47]. Students’ outlet choices in this study usually involved the relative assessment of all potential options against their key criteria. Our analysis found food outlet spatial accessibility was a recurrently reported factor, crucial to student food outlet decisions. This is reflected in wider literature [43–45]. A systematic review found a balance of evidence suggesting that spatial availability and accessibility were associated with behaviour, nutrition, and health outcomes in LMIC settings [1], in line with previous evidence mainly from HICs [48]. This association is supported by longitudinal results from Western Australia examining behaviour after residential relocation [49]. Other research also suggests that individuals from low income households are more susceptible to using unhealthy food outlets when they are near the home [50].

In Ghana and South Africa a study mapping the local foodscape, suggested that the availability of healthy neighbourhood food outlets did not reflect in the food products available in the household, highlighting the potential role of other factors [51]. Vehicle ownership and/or access to public or other transport vehicles, for instance, make it convenient for people to access outlets beyond their immediate foodscape, increasing their potential food outlet options [49, 52]. Based on this analysis, it appears that time-demands and limited access to transportation put students in a position that makes spatial accessibility a key motivation to food outlet choice. Consistent with the current study findings, other studies confirm the importance of convenience to students’ food-related choices due to exams and other academic demands [18, 44]. The emergence of online food delivery services could improve availability of healthy food, or conversely may improve the availability of unhealthy food. Many students may currently be unable to afford the added costs of using such services, but this may change in the future.

Another prominent finding is that emerging adults adapted their food outlet choices to their financial constraints. They matched cost of food at various outlets (environmental level) with their individual budgets (personal level). Consistent with other recent studies [43, 53], a mismatch between individual budgets and food prices (as an environmental factor) influences students’ decision to settle on food outlets offering cheaper food options. The mismatch impels young people to focus on the need for quantity and satiety as found for adolescents in Coventry, U.K. [54]. Even though cheaper food options per-se may not necessarily be unhealthy [42, 55], there is overwhelming evidence suggesting that cheap foods are generally not healthy or sustainable with healthy foods found to be largely expensive [56] and especially in LICs [57]. In this study, cheap outlets were reported to be unhealthy—offering high-fat and calorie-dense food options.

Hygiene or sanitation at food outlets was also a key factor emerging adults considered in their food outlet choices, although not always driving the final decision. Similar concerns have been reported by non-student Ghanaians and Kenyans living in deprived neighbourhoods [58]. In contrast to findings on concerns about long-term health issues like hypertension and cardiovascular disease reported in previous research [59, 60], the short-term health concerns like diarrhoea appeared to be more salient in this population. Interestingly or ironically, the two most patronized food outlets were associated with all the sanitation issues reported by participants. There were no other outlets within the University foodscape offering food at same (affordable) prices or offering the variety that these two did. As utility maximizers, in most cases, students are pushed to patronize the best available option after evaluating options against their relevant criteria.

Similar to what others have reported, social relationships and social interactions featured in food outlet decisions. For example, while a “noisy” informal African atmosphere was preferred for a regular social eating with friends, the same was considered a misfit for birthday parties. The atmosphere/ambience at restaurants has been found to significantly influence customer intention to patronise [61–63]. On the other hand, food outlets and food-related behaviours have been found to stimulate social relations and cooperation between groups [64, 65], within groups (as in a nuclear family [66] and between colleagues/ mates (including schoolmates, couples in romantic relationships, work colleagues [65, 67–69]. These suggest that food outlet/procurement and other food-related choices shape and strengthen social bonding, social relationships, and interactions. In this study, emerging adults used food outlets as sites for social interactions and bonding with their mates and/or friends as they engaged in various forms of social eating. One implication of this, along with the finding on budget and price, is that the university foodscape may shape and reinforce social class polarization considering students who cited that certain outlets are “out of their league.”

## Strengths and limitations

The use of FGDs can be considered a strength of this study. These support the creation of shared understandings and are credited with a greater ability for collecting “well-grounded, rich descriptions and explanations of processes in identifiable local contexts.” [70]. FGDs are thus particularly useful in research aimed at understanding how the food environment influences food behaviours of young adults.

In addition, offering participants an option between FGDs and BFPI increased opportunities for greater participation which allowed the collection of a wider range of views. Individuals who would be uncomfortable in the presence of ‘strangers’ had the option to participate with their best friend. To minimise researcher bias in analysis a second author (OO) checked the coding framework and importantly, participants were invited to comment on whether findings represented the views shared during interview and FGD sessions.

This study was limited to emerging adult participants of one university in Ghana. Findings may therefore not be indicative of the views of the wider population. However, the focus on emerging adults in this study addressed the need for research to inform tailored interventions for this specific population.

Finally, sample size adequacy may have been limited due to the sampling approach adopted. Certain subgroups (e.g., foreign/international students) were lacking from the sample. However, a good diversity of participants across disciplines, level of study and socio-demographic variables, as observed from participant characteristics reported in the results.

## Conclusion

Food consumption has important impacts on personal health and the environment, and food outlet choice is a key antecedent to food acquisition and consumption. The food outlet choice decisions in this emerging adult group were the net results of a complex interplay between intrapersonal, societal, and environmental factors with price, spatial accessibility, budget, and quantity/satiety some of the most important considerations for students. Multi-component interventions that combine structural level interventions in food retailing along with individual level components may be effective at changing emerging adult consumption behaviour in SSA, although this needs to be studied.

## Data Availability

The datasets used in this study, except identifiable personal information, are available from the corresponding author on reasonable request.
